# A RING-Type E3 Ubiquitin Ligase, *OsGW2,* Controls Chlorophyll Content and Dark-Induced Senescence in Rice

**DOI:** 10.3390/ijms21051704

**Published:** 2020-03-02

**Authors:** Kyu-Chan Shim, Sun Ha Kim, Yun-A Jeon, Hyun-Sook Lee, Cheryl Adeva, Ju-Won Kang, Hyun-Jung Kim, Thomas H Tai, Sang-Nag Ahn

**Affiliations:** 1 Department of Agronomy, Chungnam National University, Daejeon 34134, Korea; zktnrl@naver.com (K.-C.S.); sunha82@cnu.ac.kr (S.H.K.); jya0911@cnu.ac.kr (Y.-A.J.); leehs0107@gmail.com (H.-S.L.); ccadeva_758@yahoo.com (C.A.); 2 Department of Southern Area Crop Science, National Institute of Crop Science, RDA, Miryang 50424, Korea; kangjw81@korea.kr; 3 LG Chemical, Ltd., Seoul 07796, Korea; hk269@cornell.edu; 4 USDA-ARS Crops Pathology and Genetics Research Unit, Davis, CA 95616, USA; thomas.tai@usda.gov; 5 Department of Plant Sciences, University of California, Davis, CA 95616, USA

**Keywords:** leaf senescence, rice, quantitative trait loci, transcriptome analysis

## Abstract

Leaf senescence is the final stage of plant development. Many internal and external factors affect the senescence process in rice (*Oryza sativa* L.). In this study, we identified *qCC2*, a major quantitative trait locus (QTL) for chlorophyll content using a population derived from an interspecific cross between *O. sativa* (*cv*. Hwaseong) and *Oryza grandiglumis*. The *O. grandiglumis* allele at *qCC2* increased chlorophyll content and delayed senescence. *GW2* encoding E3 ubiquitin ligase in the *qCC2* region was selected as a candidate for *qCC2*. To determine if *GW2* is allelic to *qCC2*, a *gw2*-knockout mutant (*gw2-ko*) was examined using a dark-induced senescence assay. *gw2-ko* showed delayed leaf senescence in the dark with down-regulated expression of senescence-associated genes (SAGs) and chlorophyll degradation genes (CDGs). The association of the *GW2* genotype with the delayed senescence phenotype was confirmed in an F_2_ population. RNA-seq analysis was conducted to investigate 30-day-old leaf transcriptome dynamics in Hwaseong and a backcross inbred line—CR2002—under dark treatment. This resulted in the identification of genes involved in phytohormone signaling and associated with senescence. These results suggested that transcriptional regulation was associated with delayed senescence in CR2002, and RING-type E3 ubiquitin ligase GW2 was a positive regulator of leaf senescence in rice.

## 1. Introduction

Chlorophyll (Chl) is a photosynthetic pigment that is an essential component of the plant photosystem. It changes solar energy to chemical energy. Because of its photosynthetic ability, increasing Chl content in crops may be an effective way to increase grain yield and biomass production [1]. A number of studies have demonstrated that Chl content is controlled by quantitative trait locus (QTL) in various genetic backgrounds in rice [2,3,4,5,6]. 

Senescence or biological aging is the final stage of plant development [7]. During senescence, leaf color turns from green to yellow because of chlorophyll degradation [8]. Leaf yellowing is frequently used as a senescence indicator. Stay-green (non-yellowing) mutants maintain leaf greenness after the grain-ripening stage [9]. Stay-green mutants exhibit delayed leaf senescence and have been found in various plant species [8,10]. In rice, several stay-green genes have been cloned and characterized, including *Stay-Green Rice* (*SGR*), *NON-YELLOW COLORING 1* (*NYC1*), *NYC1-LIKE* (*NOL*), *Oryza sativa NAC-like, activated by Apetala3/Pistillata* (*OsNAP*) [10,11,12,13,14]. *SGR,* a highly conserved senescence-associated gene, encodes a novel chloroplast protein, and the expression of *SGR* is up-regulated in both natural and dark-induced senescence. This gene interacts with the light-harvesting chlorophyll-binding protein (LHCP) [10,14]. *NYC1* and *NOL* encode short-chain dehydrogenase/reductase (SDR) [11,12]. These two genes are co-localized in the thylakoid membrane and function as a chlorophyll *b* reductase. *OsNAP* contains a typical NAC structure at the N terminus [13]. This gene plays a role in regulating leaf senescence and acts as a key component, linking ABA signaling in rice. These genes have been used as ideal markers for the onset of the senescence process.

Ubiquitination is the post-translational modification of protein substrates [15]. The ubiquitin-proteasome pathway is known to play an important role in plant seed and organ size determination, such as *DA1*, *DA2*, *EOD1/BB*, *SAMBA*, and *SOD/UBP15* genes in *Arabidopsis* [16,17,18,19,20]. A major function of E3 ubiquitin ligase is to regulate polyubiquitination and to degrade their target substrate proteins. E3 ubiquitin ligase *BigBrother* (*BB*) controls final organ size and seed size acting in parallel with the *DA1* gene in *Arabidopsis,* and these two genes also positively control leaf senescence [17,19,20]. Delayed senescence has been confirmed by measuring the expression of *AHK3*, *CRF6*, and *ARF2*. The negative regulators of senescence—*AHK3* and *CRF6*—are expressed higher in *da1-1_bb/eod1-2* leaves, while the auxin repressor *ARF2* is expressed at a lower level than wild type [19]. *DA2,* an ortholog of *OsGW2*, controls seed and organ size by interacting with *DA1* [18].

*OsGW2*, a QTL on chromosome 2, controls grain width and weight in rice and encodes RING-type E3 ubiquitin ligase [21]. This gene might function in the degradation by the ubiquitin-proteasome pathway, and loss-of-function in *OsGW2* increases cell numbers and grain size [21]. The *OsGW2* mutant also shows increased transcript levels of *OsPCR1* during the developing grain stage, leading to an increase in Zn concentration in the seed [22]. Yeast two-hybrid and in vitro pull-down assays have shown that OsGW2 directly interacts with expansin-like 1 (EXPLA1), chitinase 14 (CHT14), and phosphoglycerate kinase (PGK) [23,24]. Transcription activation activity has been found in the *GW2*-C terminus (205 to 260) [24]. Together, the findings that OsGW2 interacts with various proteins and that the mutation of *OsGW2* has a pleiotropic effect in rice raises the possibility that *GW2* is also involved in regulating leaf senescence.

The objective of this study was to identify QTL controlling chlorophyll content and leaf senescence and to characterize candidate genes associated with this trait. *qCC2*, a major QTL for chlorophyll content, was mapped using an introgression line, CR2002, developed from an interspecific cross between *O. sativa cv*. Hwaseong and the wild species *O. grandiglumis.* CR2002 harboring *qCC2* from *O. grandiglumis* showed delayed leaf yellowing and a higher *Fv*/*Fm* value than Hwaseong. Endogenous expression levels of senescence-associated genes (SAGs) and chlorophyll degradation genes (CDGs) in CR2002 were lower than in Hwaseong. To determine if *GW2* is allelic to *qCC2* and thereby associated with delayed senescence, a *gw2*-knockout mutant (hereafter termed *gw2-ko*) was examined using dark-induced senescence (DIS) assay. *gw2*-*ko* showed delayed leaf senescence under dark conditions with down-regulated expression of SAGs and CDGs. Delayed senescence was confirmed by segregation analysis in the F_2_ generation. Comparative RNA-seq analysis was conducted to identify differentially-expressed transcripts, using 30-day-old leaves from Hwaseong and CR2002 that were subjected to dark treatment. This enabled the identification of which genotype-specific expressions were enriched in CR2002 and reduced in Hwaseong and vice versa, including genes involved in phytohormone biosynthesis and signaling, NAC transcription factors, and senescence-associated genes. Collectively, the results of this study suggested that *OsGW2* controlled chlorophyll content and was a positive regulator of leaf senescence in rice.

## 2. Results

### 2.1. QTL Analysis for Chlorophyll Content

**Figure 1:**
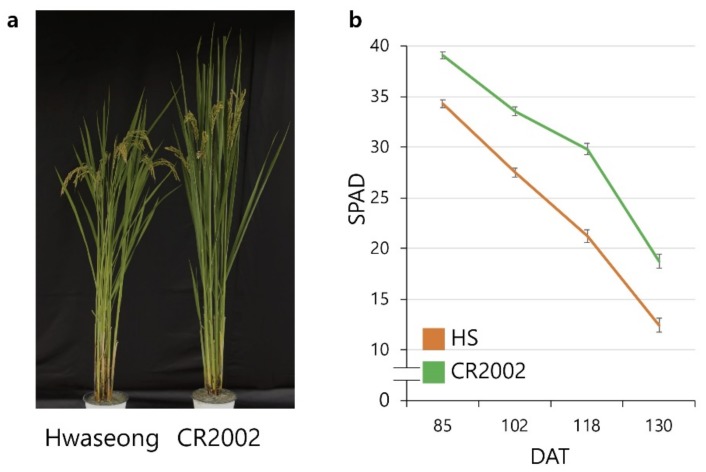
CR2002 showed a higher SPAD value and delayed senescence than Hwaseong in the field condition. Comparison of (**a**) plant morphology and (**b**) SPAD value (*n* = 30) of two parents. Error bars indicate the standard error. DAT: days after transplanting.

CR2002 exhibited a stay-green phenotype and a higher SPAD value (a parameter of leaf greenness) than Hwaseong (Figure 1b). CR2002, which had four *O. grandiglumis* introgressions, also showed differences in several agronomic traits, including grain weight (Table 1, Figure 1a, and Figure S1). To identify loci associated with chlorophyll content and the stay-green phenotype, QTL analysis was conducted using F_3_ and F_4_ populations derived from a cross between Hwaseong and CR2002. Chlorophyll contents were measured at the heading stage and one month after heading. A total of three significant QTLs located on chromosomes 1, 2, and 6 were detected in the F_3_ and F_4_ populations (Table 2 and Figure S2). A QTL for chlorophyll content (*qCC2*) was located on chromosome 2 between RM12813 and RM12983. This QTL was repeatedly detected in F_3_ and F_4_ generations at both stages, heading (Chlorophyll I) and one month after heading (Chlorophyll II). *qCC2* explained 24.6% of the phenotypic variation, and the *O. grandiglumis* allele contributed to the increased chlorophyll content, indicating that *qCC2* was a major QTL responsible for leaf greenness at heading and ripening stage.

The *qCC2* region was confirmed and fine-mapped by substitution mapping (Figure S3). SPAD values were measured four times, from 95 to 125 days after transplanting (DAT), for four substitution lines and two parental lines. CR7036 and CR7039 showed significantly higher SPAD values at 125 DAT than CR7038 and CR7058, suggesting that *qCC2* was located in the marker interval between RM3390 and RM7288 (about 1.4 Mbp). 

### 2.2. Dark-Induced Senescence in Hwaseong and CR2002

**Figure 2:**
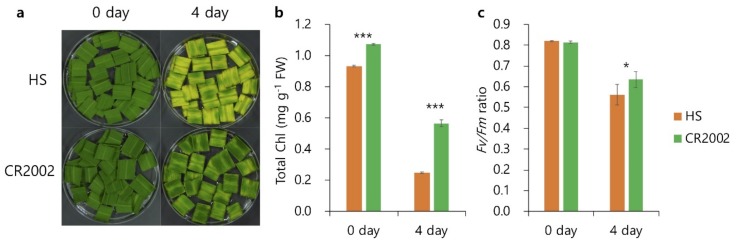
CR2002 showed delayed senescence under dark-induced senescence (DIS) conditions. Hwaseong and CR2002 were grown in a paddy field, and fully expanded flag leaves at the heading stage were used for DIS. (**a**) Detached leaves were incubated in 3 mM MES buffer (pH 5.8) at 27 °C under dark conditions. (**b**,**c**) Total chlorophyll contents (*n*= 6) and *Fv*/*Fm* ratio (*n*= 5) were compared between Hwaseong and CR2002. Error bars indicate a standard error, and more than three samples were used for each experiment. * and *** indicate significant difference at *p* < 0.05 and *p* < 0.001 based on Student’s *t*-test, respectively.

To determine whether *qCC2* is associated with senescence under dark condition, detached leaves of Hwaseong and CR2002 were incubated on 3 mM MES buffer at 27 °C under complete darkness (Figure 2). Hwaseong leaves turned yellow four days after incubation (DAI), while CR2002 remained green (Figure 2a). Although the chlorophyll content of CR2002 was higher than Hwaseong at 0 DAI, a larger difference was observed at 4 DAI between CR2002 and Hwaseong (Figure 2b). The efficiency of photosystem II (*Fv*/*Fm* ratio) of CR2002 was significantly higher than Hwaseong, indicating CR2002 showed delayed senescence in the DIS assay (Figure 2c).

**Figure 3:**
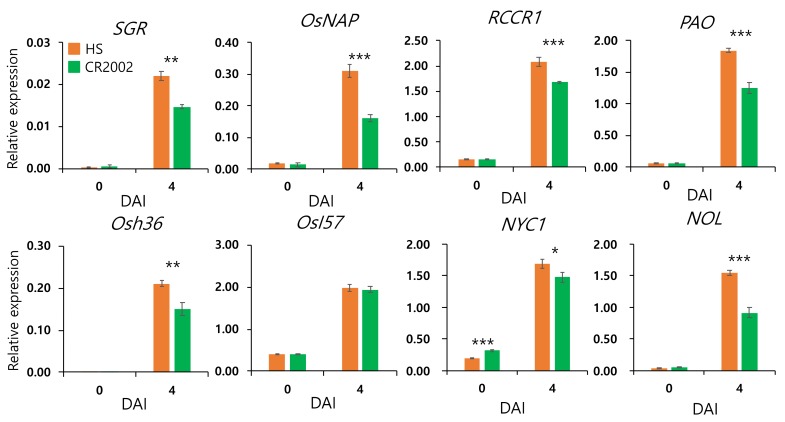
Expression of senescence-associated genes and chlorophyll degradation genes in Hwaseong and CR2002. qRT-PCR was conducted to determine the transcript level of genes. *OsUBQ5* was used for normalization. Error bars indicate the standard deviation of three replications. *, **, and *** indicate significant difference at *p* < 0.05, *p* < 0.01, and *p* < 0.001 based on Student’s *t*-test, respectively. DAI: days after incubation.

To further examine the different senescence of Hwaseong and CR2002, transcript levels of SAGs (*OsNAP*, *Osh36,* and *OsI57*) and CDGs (*SGR*, *RCCR1*, *PAO*, *NYC1*, and *NOL*) were measured by qRT-PCR using detached leaf samples (Figure 3). During DIS, transcription levels of the SAGs and CDGs showed increases of 4.9-fold to 325-fold in Hwaseong, whereas increases of 4.7-fold to 200-fold were observed in CR2002. Transcript levels of SAGs and CDGs in CR2002 were significantly lower than Hwaseong at 4 DAI, with the exception of *OsI57*, indicating that the lower expression of SAGs and CDGs in CR2002 might result in the delayed senescence exhibited by CR2002 in the DIS assay.

### 2.3. Characterization of gw2-ko

The *O. grandiglumis* segment harboring *qCC2* is approximately 1.4 Mb in size. In this region, *GW2* was selected as a candidate gene based on its function as a RING-type E3 ubiquitin ligase. A 1-bp deletion at position 316 in the coding region of the *O. grandiglumis GW2* allele, which causes a premature stop and leads to an increased grain width and thickness, was also observed in this study (Figure S4) [21]. In *Arabidopsis*, ubiquitin-activated peptidase DA1 regulates leaf senescence together with E3 ubiquitin ligase BB [18]. In addition, *DA1* controls seed and final organ size with interacting partner *DA2,* which is orthologous to *OsGW2*. Due to the previously identified pleiotropic effect of *GW2*, the *GW2* gene was selected as a candidate gene for the *qCC2* QTL.

**Figure 4:**
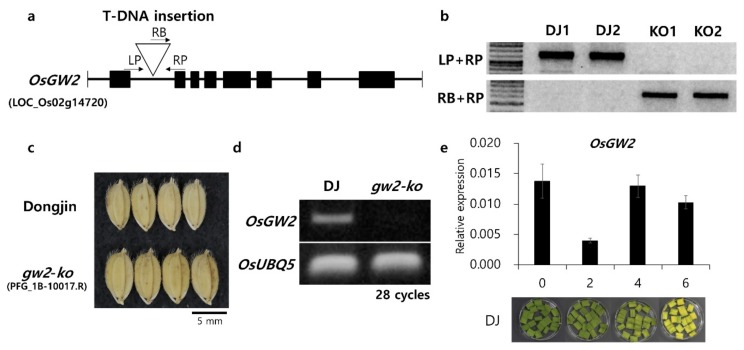
*OsGW2* T-DNA insertion knock-out mutant (*gw2-ko*) in wild-type Dongjin genetic background. (**a**) Schematic diagram showing the location of T-DNA insertion of the PFG_1B-10017.R mutant. (**b**) T-DNA insertion was confirmed using two sets of primers indicated in (**a**), and lines homozygous for the T-DNA insertion were used for this experiment. (**c**) Comparison of grain shape between wild type and *gw2-ko*. (**d**) Confirmation of *OsGW2* knock-out by semi-quantitative RT-PCR. *OsUBQ5* was used as a loading control. (**e**) Gene expression pattern of *OsGW2* during the dark-induced senescence. The transcript level of genes was determined by qRT-PCR. *OsUBQ5* was used for normalization.

To determine if *GW2* is associated with delayed senescence, a T-DNA insertional mutant (PFG_1B-10017.R) was selected from a T-DNA insertional mutant library [25]. The T-DNA insertion was confirmed by two sets of PCR primers, and homozygous T-DNA lines were selected by genomic PCR (Figure 4a,b). The *OsGW2* knock-out mutant (*gw2-ko*) showed increased grain size, and the transcription of *OsGW2* in wild type (WT Dongjin) and *gw2-ko* was evaluated using semi-quantitative RT-PCR (Figure 4c,d). The *OsGW2* transcript level was examined during DIS in WT. The expression level was down-regulated at 2 DAI, whereas gene expression was up-regulated after 4 DAI, and a similar level of gene expression with 0 DAI was observed (Figure 4e). Consistent expression patterns of *OsGW2* and *OgGW2 (O. grandiglumis GW2)* were found in Hwaseong and CR2002, but CR2002 showed lower transcript levels than Hwaseong at 0 and 4 DAI (Figure S5).

**Figure 5:**
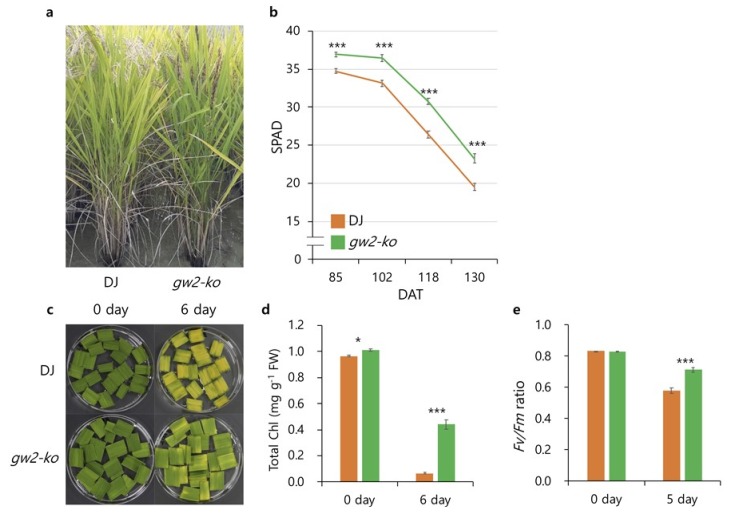
*gw2-ko* mutant showed delayed senescence. Comparison of (**a**) plant morphology (at 138 days after transplanting) and (**b**) SPAD value (*n* = 30) of wild-type Dongjin and *gw2-ko*. (**c**) Wild type and *gw2-ko* were grown in a paddy field, and fully expanded flag leaves at the heading stage were used for DIS. (**d**,**e**) Total chlorophyll contents (*n* = 6) and *Fv*/*Fm* ratio (*n* = 10) were compared between wild type and *gw2-ko*. Error bars indicate a standard error, and more than six samples were used for each experiment. * and *** indicate significant difference at *p* < 0.05 and *p* < 0.001 based on Student’s *t*-test, respectively.

The *gw2-ko* mutant showed significantly higher SPAD values than Dongjin from 85 to 130 DAT under the field conditions (Figure 5a,b). To characterize the phenotypic difference between Dongjin and *gw2-ko* during DIS, fully expanded detached leaves were incubated in 3 mM MES buffer (pH 5.8) at 27 °C in the dark (Figure 5c). Leaves of Dongjin turned yellow at 6 DAI, while *gw2-ko* remained green. Total chlorophyll contents of Dongjin and *gw2-ko* at 6 DAI were also significantly different, despite the total chlorophyll contents of *gw2-ko* at 0 DAI being slightly higher than Dongjin (Figure 5d). The *Fv*/*Fm* ratio remained higher in *gw2-ko* than Dongjin at 5 DAI (Figure 5e). These results demonstrated that leaf senescence was delayed in *gw2-ko*.

**Figure 6:**
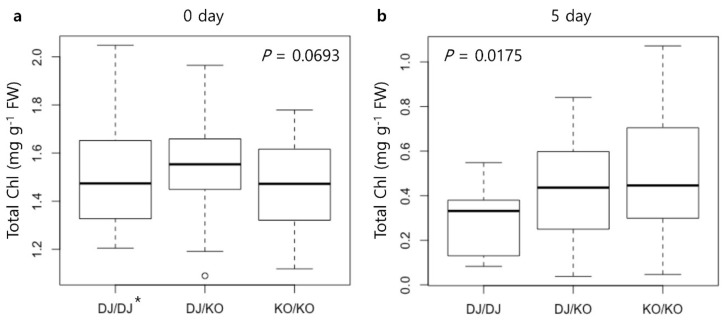
Boxplot of chlorophyll contents at (**a**) 0 day after incubation and (**b**) 5 days after incubation under the dark-induced senescence conditions in the F_2_ population derived from a cross between Dongjin and *gw2-ko*. Genotype was determined with two sets of primers, as indicated in Figure 4a—LP+RP and RB+RP. One-way ANOVA was conducted to determine the significant difference between genotypes. *DJ/DJ: homozygous for Dongjin, DJ/KO: heterozygous, KO/KO: homozygous for *gw2-ko*.

An F_2_ population (*n* = 107) derived from a cross between Dongjin and *gw2-ko* was evaluated for DIS to determine if the T-DNA insertion in *OsGW2* was responsible for delayed senescence. After five days of dark incubation, plants homozygous for *gw2-ko* genotype (KO/KO group) showed significantly higher total chlorophyll contents than those homozygous for Dongjin genotype (DJ/DJ group) (Figure 6a,b). This segregation analysis suggested that T-DNA insertion in *OsGW2* caused the delayed senescence exhibited by *gw2-ko*.

**Figure 7:**
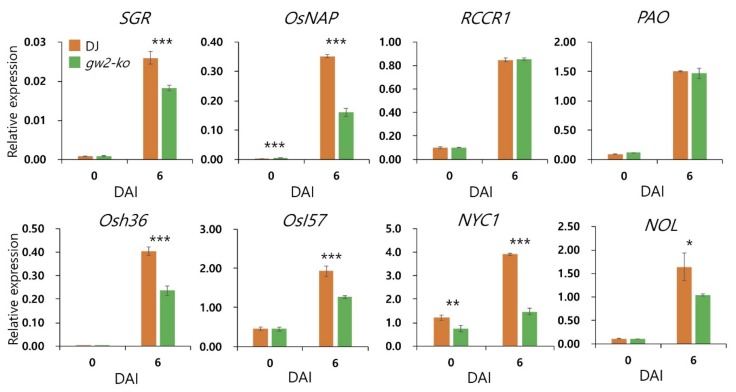
Expression of senescence-associated genes and chlorophyll degradation genes compared in Dongjin and *gw2-ko*. qRT-PCR was conducted to determine the transcript level of genes. *OsUBQ5* was used for normalization. Error bars indicate the standard deviation of three replications. *, **, and *** indicate significant difference at *p* < 0.05, *p* < 0.01, and *p* < 0.001 based on Student’s *t*-test, respectively. DAI: days after incubation.

Endogenous expression levels of SAGs and CDGs were examined (Figure 7). Among CDGs, *SGR*, *NYC1*, and *NOL* were significantly down-regulated in *gw2-ko* at 6 DAI, but *PAO* and *RCCR1* showed no significant difference between Dongjin and *gw2-ko*. SAGs (*OsNAP*, *Osh36*, and *OsI57*) were also down-regulated in *gw2-ko*. The loss-of-function of *GW2* led to the down-regulation of SAGs and CDGs during DIS.

The physiological phenotypes and endogenous transcript levels during DIS indicated that *gw2-ko* showed delayed leaf senescence compared to wild type and that *GW2* positively regulated leaf senescence in rice.

### 2.4. Transcriptome Analysis using Hwaseong and CR2002

To gain a better understanding of the mechanism for delayed senescence under dark-treatment conditions, the transcriptome of leaves from Hwaseong and CR2002 plants before (control) and after dark-treatment for 3 days were investigated by RNA-seq. A total of 264 million reads were generated using the Illumina Hiseq 4000 platform, and adapter and low-quality read trimming were conducted (Table S2). A total of 262 million high-quality reads were mapped against the reference genome sequence, and an average mapping rate of 66% was observed (Table S2). A multi-dimensional scaling plot showed that three replicates were clustered, indicating samples were consistently generated (Figure S6).

**Figure 8:**
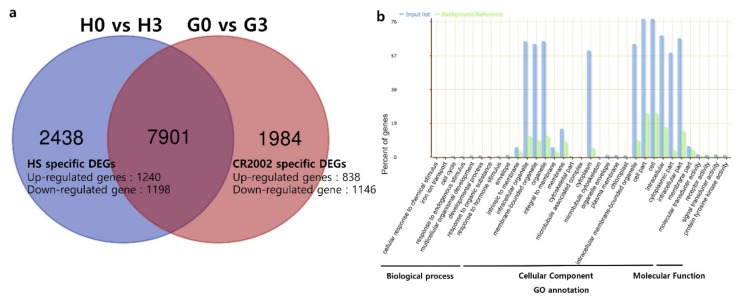
Summary of the numbers of differentially expressed genes (DEGs) upon incubation of leaves of two lines (Hwaseong and CR2002) in complete darkness. (**a**) A Venn diagram, showing the number of genes shared and distinct to each genotype. (**b**) Significantly enriched gene ontology (GO) terms for CR2002-specific DEGs. Blue and green bars indicate the input list of CR2002 and background/reference, respectively. H0, H3, G0, and G3 indicate Hwaseong day 0, Hwaseong day 3, CR2002 day 0, and CR2002 day 3 in dark condition, respectively.

Differentially expressed genes (DEGs) were identified in Hwaseong and CR2002 under dark treatment compared with control conditions. DEGs were determined based on the criteria of a greater than 2-fold change with significance at Benjamini-Hochberg false discovery rate adjusted *p* < 0.05. A total of 10,339 DEGs was detected in the comparison of 0 day and 3 days of dark treatment in Hwaseong, and 9885 DEGs were found in CR2002 genotype during the dark treatment (Figure 8a). A Venn diagram of the DEGs indicated that Hwaseong and CR2002 shared 7901 of them (Table S5). Among these 7901 genes, only 29 genes showed different regulation patterns between Hwaseong and CR2002. For example, Os09g0511600 was up-regulated in Hwaseong by 5.13 log_2_FC (fold-change), while down-regulated in CR2002 by -8.26 log_2_FC. In addition, a total of 2438 and 1984 genes were specifically identified as DEGs in Hwaseong and CR2002, respectively (Tables S3 and S4). For Hwaseong, 1240 and 1198 genes were specifically up- and down-regulated during the dark treatment, respectively (Figure 8a and Table S3). A total of 838 up-regulated genes and 1146 down-regulated genes were specifically identified during the dark treatment in CR2002 (Figure 8a and Table S4). 

To know functional information of the DEGs specifically detected in Hwaseong and CR2002, gene ontology (GO) analysis was performed with agriGO v2.0. For the 1984 CR2002-specific DEGs, a total of 33 GO terms was significantly enriched (Figure 8b). Among 33 GO terms, biological process, cellular component, and molecular function ontologies included 8, 21, and 4 GO terms, respectively. For Hwaseong-specific DEGs, a total of 33 GO terms were also significantly overrepresented. Of these GO terms, 6, 23, and 4 were included in the biological process, cellular component, and molecular function ontologies, respectively (Figure S7). Hwaseong-specific DEGs included intracellular signaling cascade, organelle membrane, cytosol, cytoskeleton, Golgi membrane, endomembrane system, and Golgi apparatus GO terms, while iron ion transport, cytoplasmic part, envelope, cellular response to chemical stimulus, cell cycle, microtubule-associated complex, and organelle envelope were included only in CR2002-specific DEGs.

To understand the mechanism involved in delayed senescence in CR2002, the SAGs, CDGs, and genes involved in phytohormone biosynthesis and signaling were examined from the DEGs. A total of 109 genes were identified (Table S6). Among these genes, we focused on those specifically detected in Hwaseong or CR2002 (Table 3). During the dark treatment, *OsNIT1*, *OsDWF1*, and *OsRR3* were up-regulated in CR2002, and *OsERF5*, *OsABI1*, *OsJAZ1*, and *OsGLU* were down-regulated. Phytohormone signaling and biosynthesis genes were mainly found in the CR2002 DEGs. When these seven DEGs were subjected to RT-qPCR using the same G0/G3 samples as for RNA-Seq, similar expression patterns were observed, suggesting the involvement of these DEGs in delayed senescence in CR2002 (Figure S8). For Hwaseong, 18 genes were identified from the DEGs, and 10 and 8 genes were down- and up-regulated, respectively. Hwaseong-specific DEGs included phytohormone biosynthesis and signaling genes, NAC transcription factors, and senescence-associated genes.

## 3. Discussion

### 3.1. qCC2 is Associated with Chlorophyll Content and Leaf Senescence

Chlorophyll content, one of the most important traits due to its role in photosynthesis, is regulated by polygenic loci. Several studies have identified a number of QTLs across various genetic resources and environments [3,4,5,6]. A number of studies reported QTLs on the short arm of chromosome 2. Four QTLs have been mapped for stay-green traits on chromosome 2 between RM145 and RM322 using 190 doubled haploid lines from a cross between ‘Zhenshan 97′ and ‘Wuyujing 2′ [2]. Jiang et al. (2012) reported QTL for chlorophyll content from heading to the maturity stage (*qCHM2*) located on the short arm of chromosome 2 between RM145 and RM29 [5]. In this study, three QTLs for chlorophyll content were detected using progeny from an interspecific cross between *O. sativa* and *O. grandiglumis*. *qCC2* was located on chromosome 2 between RM3390 and RM7288, which were approximately 1.4 Mbp apart. This QTL was detected in F_3_ and F_4_ generations and at two different developmental stages, respectively, and confirmed by substitution mapping (Figure S3). *qCC2* shared a similar location with QTLs reported in previous studies [2,5]. Although allelism tests are needed to clarify the relationships among these QTLs, these studies indicated the presence of genes associated with chlorophyll content and leaf senescence on the short arm region of chromosome 2. 

When Hwaseong and CR2002 were tested for dark-induced senescence, CR2002 showed higher chlorophyll content with delayed leaf senescence than Hwaseong (Figure 2a,b). *Fv*/*Fm* values were also significantly different at 4 days after incubation (Figure 2c). Endogenous expression levels of SAGs and CDGs were severely down-regulated in CR2002 than Hwaseong, and these results indicated that *qCC2* was associated with leaf senescence in CR2002.

### 3.2. GW2 Positively Regulates Leaf Senescence

*GW2* encodes a RING-type E3 ubiquitin ligase and resides in the *qCC2* region (1.4 Mbp). The *O. grandiglumis GW2* allele in CR2002 has the 1-bp deletion in 4th exon, which causes a premature stop and truncated protein (Figure S4) [21]. Many studies have suggested ubiquitination as a candidate pathway for the regulation of senescence. *SAUL1* encodes E3 ubiquitin ligase, which is required for suppression of premature senescence in *Arabidopsis* [26]. Also, *spl11* encodes E3 ubiquitin ligase, which regulates cell death in rice [27]. E3 ubiquitin ligase *Big Brother* (*BB*) gene and ubiquitin receptor *DA1* gene negatively regulate leaf size and promote senescence [19]. Mutations in *DA1* and *BB* enhance leaf growth, an effect that is synergistically increased in the double mutant. A *da1-1/eod1-2* double mutant especially exhibits a longer lifespan than wild type or the single mutants [19]. In addition, DA1 physically interacts with DA2, an ortholog of *GW2*, and *DA2* acts synergistically with *DA1* to regulate seed size in *Arabidopsis* [18]. Based on these previous results, *GW2* was chosen as a candidate gene for the delayed senescence phenotype in CR2002. The transcript level of *GW2* was down-regulated at 2 DAI and showed a similar expression level at 0 DAI and at 4 and 6 DAI in the dark (Figure 4e and Figure S5). This expression pattern was similar to the *NYC4* gene, which functions in the degradation of chlorophyll and chlorophyll-protein complexes during DIS [28]. The effect of *gw2-ko* was examined in DIS (Figure 5c). *gw2-ko* showed delayed senescence and down-regulated expression of SAGs and CDGs. Compared to Hwaseong and Dongjin, most of the SAGs and CDGs genes were consistently down-regulated in CR2002 and *gw2-ko* during DIS. However, transcript levels of *RCCR1* (*red chlorophyll catabolite reductase 1*), *PAO* (*Pheophorbide a oxygenase*), and *OsI57* (*putative 3-ketoacyl-CoA thiolase*) did not show significant differences between Dongjin and *gw2-ko* or Hwaseong and CR2002 in DIS. These results suggested that *RCCR1* and *PAO* might function down-stream in the chlorophyll degradation pathway [8]. Because the transcript levels of *RCCR1* and *PAO* in *gw2-ko* were not significantly different from wild-type Dongjin, *GW2* might affect genes up-stream in the chlorophyll degradation pathway.

### 3.3. Differentially Expressed Genes Associated with Leaf Senescence

Comparative RNA-seq analysis was conducted to identify differentially-expressed transcripts, using 30-day-old leaves from Hwaseong and CR2002 to gain a better understanding of the mechanism involved in delayed senescence. A total of 10,339 and 9885 DEGs were identified in Hwaseong and CR2002 under dark treatment compared with the control, respectively (Figure 8a). To examine the difference between Hwaseong and CR2002, Hwaseong- and CR2002-specific DEGs were extracted. In CR2002-specific DEGs, 33 GO terms were enriched, and these included iron ion transport, response to hormone stimulus, cell cycle, response to endogenous stimulus, and chloroplast. Transporter genes are closely associated with leaf senescence process. During senescence, remobilization of nutrients from senescing cells to developing tissues is mediated by transporters, and transporter genes show notable changes in expression [13,29]. Chloroplast GO term contains 15 CR2002-specific DEGs, including Os07g0462000 (*OsSG1*), Os07g0558500 (*NYC4*), Os02g0152400 (*RbcS1*) [28,30]. Os07g0462000 encodes glutamate-cysteine ligase and possibly controls chlorophyll content and stay-green phenotype [30]. Os07g0558500 (*NYC4*) is an ortholog of *THF1* in *Arabidopsis.* The *AtTHF1* is known as a multi-function protein involved in acclimation to high light, sugar sensing, and disease resistance, while *nyc4-1* mutant shows the stay-green phenotype in rice [28]. Os02g0152400 encodes the rubisco small subunit 1, which reflects involvement in the regulation of rubisco catalytic activity.

Transcription factors (TFs) play an important role in regulating leaf senescence. NAC (NAM, ATAF1, and CUC2) transcription factor family is one of the large TF families, and many NAC TFs are up-regulated during leaf senescence [31]. In addition, several NAC TFs regulating leaf senescence have been characterized in various plant species [13,32,33,34,35,36]. Transcript levels of NAC transcription factors (*OsNAP, ONAC120*, and *ONAC122*) were specifically up-regulated in Hwaseong (Table 3). *OsNAP* is a positive regulator of leaf senescence, and its orthologs *TtNAM-B1* and *AtNAP* have shown similar function in wheat and *Arabidopsis* [13,32,33]. *OsNAC122* (or *OsNAC10*, Os11g0126900) over-expression and root-specific expression transgenic have shown significantly improved drought, high salinity, and low-temperature tolerance in rice [37].

Phytohormone signaling and biosynthesis-related genes were identified in Hwaseong- and CR2002-specific DEGs. During dark treatment, ethylene (*OsERF5*), ABA (*OsABI1*), and jasmonic acid (*OsJAZ1*) signaling genes were significantly down-regulated, while auxin synthesis (*OsNIT2*), brassinosteroid biosynthesis (*OsDWF1*), and cytokinin signaling (*OsRR3*) genes were up-regulated in CR2002 (Table 3). In contrast, ethylene biosynthesis and signaling (*OsACS2* and *OsERF2*) and auxin signaling (*OsARF2*) genes were up-regulated in Hwaseong, whereas cytokinin signaling (*OsRR6*, *OsRR9*, and *OsRR10*), brassinosteroid signaling (*OsBRI1*), and *gibberellin 20-oxidase* (*OsGA20ox1*) were down-regulated (Table 3). These results indicated that delayed leaf senescence in CR2002 (*gw2-ko*) was associated with phytohormone signaling or biosynthesis pathways in rice. The rice stay-green mutant *oself3.1* has shown down-regulated transcript levels of ABA-, ethylene-, JA-associated genes in microarray analysis [7]. The results from the present study were consistent with the report that *OsELF3.1* promoted leaf senescence by modulating signaling pathways [7]. To further investigate this relationship with phytohormones, gene expression of *GW2* in response to plant hormones was examined using the RiceXPro database. *GW2* did not show large changes in response to plant hormones (data not shown) [38]. In durum wheat, a *GW2* knock-down mutant has shown enhanced *cytokinin dehydrogenase 1* (*CKX1*) transcript level and down-regulation of *cytokinin dehydrogenase 2* (*CKX2*) and *gibberellin oxidase 3* (*GA3-ox*) [39]. In addition, a wheat near-isogenic line (NIL) harboring the *TaGW2-6A* allelic variant has shown an increase in not only grain size but also endogenous cytokinin content (Z+ZR) [40]. Compared to the control (Chinese Spring), the expression level of cytokinin biosynthesis genes (*TaIPT2*, *TaIPT3*, *TaIPT5*, and *TaIPT8*) are found to be up-regulated, while cytokinin degradation genes (*TaCKX1*, *TaCKX2*, and *TaCKX6*) are found to be down-regulated in the NIL during the endosperm development [40]. These results supported the possibility that delayed senescence in CR2002 and *gw2-ko* was mediated by phytohormone-related pathways.

### 3.4. GW2 as an E3 Ubiquitin Ligase

The ubiquitination process is a post-translational modification. E3 ligases function in the regulation of polyubiquitination and are involved in target protein degradation. Several studies have reported interacting partners of GW2. Target proteins of GW2 have been revealed through yeast two-hybrid screening [24]. Yeast two-hybrid and pull-down assays have shown that expansin-like 1 (EXPLA1) directly interacts with *GW2*. In addition, in vitro assays have identified the ubiquitination of EXPLA1 by *GW2* at lysine 279 (K279). Proteomic analysis has shown that chitinase 14 (CHT14) and phosphoglycerate kinase (PGK) directly interact with GW2 [23]. However, genes that are directly associated with leaf senescence have not been reported. Identification of GW2 substrate protein(s) responsible for leaf senescence would be key to understanding the post-translational molecular mechanism of leaf senescence in rice.

## 4. Materials and Methods

### 4.1. Plant Materials

CR2002 was derived from a cross between *O. sativa* Korean elite line ‘Hwaseong’ as a recurrent parent and wild species *O. grandiglumis* (2n = 48, CCDD, Acc. No. 101154) [41]. Four *O. grandiglumis* chromosome segments were found in CR2002 on chromosomes 1, 2, 6, and 10. CR2002 was backcrossed with Hwaseong to produce an F_2_ population. A total of 705 F_2_ plants were genotyped using SSR markers from the introgression regions. Fifty-eight F_3_ plants with different combinations of *O. grandiglumis* homozygous segments were selected and advanced to the F_3_. Among the 58 F_3_ lines, 17 that have different recombination break-points within the *qCC2* region were selected and advanced to the F_4_ generation. QTL analysis was performed using two replications of the 58 F_3_ lines and 17 F_4_ lines, which were grown in the Chungnam National University paddy field during 2015 and 2016, respectively. For substitution mapping, four F_4_ recombinant lines in the *qCC2* region were investigated in 2017. To determine whether *OsGW2* is associated with leaf senescence, a *gw2*-knockout (*gw2-ko*) mutant (PFG_1B-10017.R) in the *japonica* rice ‘Dongjin’ background was used for evaluating leaf senescence [42,43]. An F_2_ population (*n* = 107) derived from a cross between Dongjin and *gw2-ko* was generated and used for DIS assay to confirm whether the T-DNA insertion in *OsGW2* locus was responsible for delayed senescence. 

### 4.2. Phenotypic Evaluation

A total of 10 traits, including chlorophyll content and three-grain morphology traits (Table 1), were evaluated in the parental lines (*n*= 10) using the methods, as described in Yoon et al. (2006) [41].

To evaluate chlorophyll content, three methods were used. First, fluorescence ratio F_735_/F_700_ was measured in F_3_ and F_4_ generation plants, and the relative chlorophyll content (mg/m^2^) was evaluated using CCM-300 (OPTI-SCIENCE, Hudson, NH, USA). This data was used for QTL analysis. Second, SPAD values were measured to compare parental lines (Hwaseong, CR2002, Dongjin, and *gw2-ko*) using a SPAD-502Plus (KONICA MINOLTA, Tokyo, Japan). Third, total chlorophyll was extracted from detached leaves and used for dark-induced senescence using pre-chilled 80% acetone [7]. The absorbance of supernatants was measured at 645 and 663 nm using a UV/VIS spectrophotometer (Hanson Tech., Seoul, Korea) [44]. For the dark-induced senescence (DIS) assay, fully expanded flag leaves were detached and placed in 3 mM MES buffer (pH 5.8) and incubated at 27 °C under complete darkness [45]. *Fv*/*Fm* ratio was measured using a Hansatech PEA MK2 (Hansatech, Norfolk, UK). The middle part of leaf samples from DIS was adapted in the dark for 30 min to complete the oxidation of Q_A_, and then the *Fv*/*Fm* value was measured. A student’s *t*-test was performed for trait comparisons.

### 4.3. DNA Extraction and QTL Analysis

DNA was extracted from leaf tissues using the extraction method, as described in Causse et al. (1994) [46]. Genotyping was conducted in 58 F_3_ and 17 F_4_ lines using 11 polymorphic SSR markers between CR2002 and Hwaseong. PCR was conducted, as described in Shim et al. (2019) [47]. PCR products were separated on 3% metaphor agar stained with StaySafe Nucleic Acid Gel Stain (RBC, Taiwan) or 4% polyacrylamide denaturing gel stained with Silver Staining Kit (Bioneer, Korea), respectively.

QTLs were determined by single-marker analysis (SMA). SMA was conducted to establish the effect of each marker on each trait. In SMA, a QTL was declared when the phenotype was associated with SSR genotype at *p* < 0.05 by one-way analysis of variance (ANOVA). The observed phenotypic variation was estimated using the coefficient of determination (R^2^). For Tukey’s test, Minitab19 software was used. Student’s *t*-test was conducted using Microsoft Excel. Boxplots were drawn using R version 3.5.3.

### 4.4. RNA Isolation and Quantitative Real-Time PCR

Total RNA of detached leaves from DIS was isolated using NucleoSpin RNA (Macherey Nagel, Deuren, Germany), according to the manufacturer’s instructions. Following reverse-transcription into the first-strand cDNA with SmartGene Mixed cDNA synthesis kit (SJ Bioscience, Daejeon, Korea), real-time PCR was performed using a CFX Connect Real-Time System (Bio-Rad, Hercules, CA, USA). Each reaction contained 10 µL of 2× SYBR Green Master Mix reagent, 2 µL of diluted cDNA samples, 1 µL of 10 pmol gene-specific primers in a final volume of 20 µL. The real-time PCR protocol was: 15 min at 95 °C to denature and activate an enzyme, followed by 40 cycles at 95 °C for 20 s (denaturation), 60 °C for 40 s (annealing), 72 °C for 30 s (extension). qRT-PCR was conducted according to the 2^-∆∆Ct^ method [48]. Rice *UBIQUITIN5* (*OsUBQ5*) was used for normalization, and relative expression levels were compared by Student’s *t*-test. Primers used in this study are listed in Table S1.

### 4.5. RNA-Seq Analysis

The 30-day-old Hwaseong and CR2002 plants were dark-treated to induce senescence for three days. From plants each before (day 0) and 3 days after dark-treatment (day 3), the 5th leaves were collected with three replicates, and total RNA was extracted, as described above. A total of 12 transcriptome libraries were constructed using TruSeq RNA Sample Prep Kit v2 (Illumina, San Diego, CA, USA), according to the manufacturer’s instructions. The library construction was conducted by GnC Bio Inc. (Daejeon, Korea). The constructed libraries were sequenced on a HiSeq 4000 instrument with 150 bp of paired-end reads. The sequence data generated from this study have been deposited at the National Center for Biotechnology Information Sequence Read Archive (SRA) under accession number PRJNA602373. Sequencing results were checked using FastQC (version 0.11.7). Raw sequences were processed to obtain clean reads by removing adapters and low-quality reads, using SCYTHE (https://github.com/vsbuffalo/scythe) and SICKLE (https://github.com/najoshi/sickle), respectively. High-quality reads were mapped on rice reference genome IRGSP-1.0, and transcript abundances were quantified using Salmon version 0.8.2 [49]. Differentially expressed genes (DEGs) were determined with more than 2-fold change with significance at Benjamini-Hochberg false discovery rate adjusted *p* < 0.05 using Limma-Voom package [50,51]. GO enrichment was carried out using agriGO v2.0 singular enrichment analysis [52].

## 5. Conclusions

A mapping population derived from an interspecific cross between *O. sativa* and *O. grandiglumis* facilitated the discovery of *qCC2*, a major QTL for chlorophyll content. The *O. grandiglumis* allele at *qCC2* increased chlorophyll content and delayed senescence. Building on several previous studies, *GW2*, which encodes E3 ubiquitin ligase, was selected as the most probable candidate gene for this phenotype, and this was confirmed through genetic analysis of a knockout mutant. The *gw2-ko* mutant showed delayed leaf senescence under dark conditions with down-regulation of SAGs and CDGs, and the delayed senescence trait completely segregated with the *gw2-ko* gene. On the basis of this work, the RING-type ubiquitin ligase *GW2* appeared to function as a positive regulator of leaf senescence in rice. This study also represented the first investigation of global gene expression in rice leaves subjected to complete darkness. The results of this whole-genome transcriptome analysis of Hwaseong and CR2002 highlighted the processes and genes that are likely to play a role in the mechanisms underlying leaf senescence in rice (e.g., carbohydrate metabolism, pyrophosphate-dependent energy conservation, and ethylene signaling). The QTL analysis and the genotype-specific regulation suggested that senescence was regulated at the transcriptional level, although the possibility of post-translational regulation by GW2 requires further investigation.

## Figures and Tables

**Table 1:** Comparison of agronomic traits and chlorophyll content between Hwaseong and CR2002.

Trait	Hwaseong	CR2002	*p*-Value
Plant height (cm)	97 ± 4.00 *	101 ± 3.84	0.000
Stem diameter (mm)	4.47 ± 0.24	5.26 ± 0.48	0.000
Panicle length (cm)	21 ± 1.28	19 ± 1.04	0.000
First internode diameter (mm)	1.72 ± 0.12	1.99 ± 0.14	0.000
Grain length (mm)	7.14 ± 0.45	7.46 ± 0.17	0.002
Grain width (mm)	3.29 ± 0.13	3.83 ± 0.10	0.000
Grain thickness (mm)	2.20 ± 0.06	2.56 ± 0.10	0.000
1000-grain weight (g)	25.8 ± 0.68	32.9 ± 1.04	0.000
Chlorophyll content I (mg/m^2^)	440 ± 16.38	457 ± 37.57	0.048
Chlorophyll content II (mg/m^2^)	181 ± 50.57	216 ± 50.86	0.038

* Data are presented as mean ± standard deviation. The student’s *t*-test was conducted for *p*-value. Chlorophyll content I and II were measured at the heading stage and 1 month after heading, respectively.

**Table 2:** QTLs (quantitative trait loci) for chlorophyll content based on one-way ANOVA in the F_3_ and F_4_.

Trait ^1^	Gen.	QTL	Chr.	Marker	*p*-Value	R^2^ (%)	H/H ^2^	G/G
Chlorophyll content I	F_3_	*qCC1*	1	RM11302-RM11315	0.002	3.48	442	452
	F_4_	*qCC2*	2	RM7288-RM12983	0.029	10.76	546	570
Chlorophyll content II	F_3_	*qCC2*	2	RM12813	0.018	10.68	173	189
	F_4_	*qCC2*	2	RM12813-RM7288	0.001	24.63	497	545
	F_3_	*qCC6*	6	RM584	0.018	10.62	454	434

^1^ Chlorophyll content I and II were measured at heading stage and heading after 1 month, respectively. ^2^ H/H and G/G indicate the mean chlorophyll contents of homozygous for Hwaseong and *O. grandiglumis* genotypes, respectively.

**Table 3:** List of differentially expressed genes (DEGs) associated with senescence, chlorophyll degradation, and phytohormone specifically detected in CR2002 and Hwaseong.

Gene	CR2002		Symbol	Description
	log_2_FC	Adj. *p*-Value		
Os07g0410300	−3.676	0.037	*OsERF5*	Conserved hypothetical protein.
Os09g0532400	−2.653	0.031	*OsABI1*	Signal transduction response regulator, receiver region domain containing protein.
Os10g0392400	−1.577	0.044	*OsJAZ1*	Tify domain containing protein.
Os07g0658400	−1.013	0.012	*OsGLU*	Ferredoxin-dependent glutamate synthase, Leaf senescence, and nitrogen remobilization.
Os02g0635200	1.081	0.000	*OsNIT1*	Similar to Nitrilase 2.
Os10g0397400	1.360	0.003	*OsDWF1*	Dim/dwf1 protein, Cell elongation protein DIMINUTO/Dwarf1, Brassinosteroid (BR) biosynthesis.
Os02g0830200	2.592	0.003	*OsRR3*	A-type response regulator, Cytokinin signaling.
Gene	Hwaseong		Symbol	Description
	log_2_FC	Adj. *p*-value		
Os11g0143300	−2.966	0.011	*OsRR9*	A-type response regulator, Cytokinin signaling.
Os12g0139400	−2.671	0.006	*OsRR10*	A-type response regulator, Cytokinin signaling.
Os11g0143200	−2.423	0.047	*OsCPD1*	Similar to Cytochrome P450 90A1.
Os03g0856700	−2.126	0.027	*OsGA20ox1*	Gibberellin 20 oxidase 1.
Os04g0673300	−1.981	0.002	*OsRR6*	A-type response regulator, Cytokinin signaling.
Os02g0164900	−1.524	0.002	*OsARF6*	Similar to Auxin response factor 3.
Os01g0208600	−1.462	0.021	*OsSCAR1*	SCAR-like protein 2, Component of the suppressor of cAMP receptor/Wiskott-Aldrich syndrome protein family verprolin-homologous (SCAR/WAVE) complex, Actin organization, Panicle development, Regulation of water loss.
Os01g0718300	−1.328	0.005	*OsBRI1*	Brassinosteroid LRR receptor kinase, Similar to Brassinosteroid-insensitive 1.
Os01g0723100	−1.323	0.001		Senescence-associated family protein.
Os03g0265100	−1.229	0	*PLS2*	Glycosyl transferase, group 1 domain containing protein.
Os07g0209000	1.096	0	*OsDGL*	Dolichyl-diphosphooligosaccharide-protein glycosyltransferase 48 kDa subunit precursor, N-glycosylation.
Os02g0324700	1.184	0.001		Similar to senescence-associated protein.
Os01g0927600	1.192	0	*OsARF2*	Similar to Auxin response factor 2 (ARF1-binding protein).
Os01g0752500	1.321	0.006	*OsERF2*	APETELA2/ethylene response factor (AP2/ERF) type transcription factor, Negative regulation of disease resistance, Negative regulation of salt tolerance.
Os03g0327800	1.872	0.013	*OsNAP*	NAC Family transcriptional activator, Abiotic stress response, Positive regulator of leaf senescence.
Os10g0477600	1.942	0.024	*ONAC120*	Similar to NAM / CUC2-like protein.
Os11g0126900	4.026	0.048	*ONAC122*	NAC-domain protein, Drought tolerance.
Os04g0578000	5.520	0.028	*OsACS2*	ACC synthase, Ethylene biosynthesis.

## Supplementary Materials

Supplementary materials can be found at https://www.mdpi.com/1422-0067/21/5/1704/s1.

## Abbreviations

DIS	Dark-induced senescence
SNP	Single nucleotide polymorphism
InDel	Insertion and deletion
qRT-PCR	Quantitative real-time PCR
SSR	Simple sequence repeat
CDG	Chlorophyll degradation gens
QTL	Quantitative trait loci
SAG	Senescence-associated gene
WT	Wild type
