# Supplementary material for: A RING-Type E3 Ubiquitin Ligase, OsGW2, Controls Chlorophyll Content and Dark-Induced Senescence in Rice
Source: Int J Mol Sci. 2020 Mar 2;21(5):1704. doi: 10.3390/ijms21051704 (PMC7084548; doi:10.3390/ijms21051704)
Supplement: Supplementary file 1 [file ijms-21-01704-s001.zip › Supplementary_figures-rev.pdf]

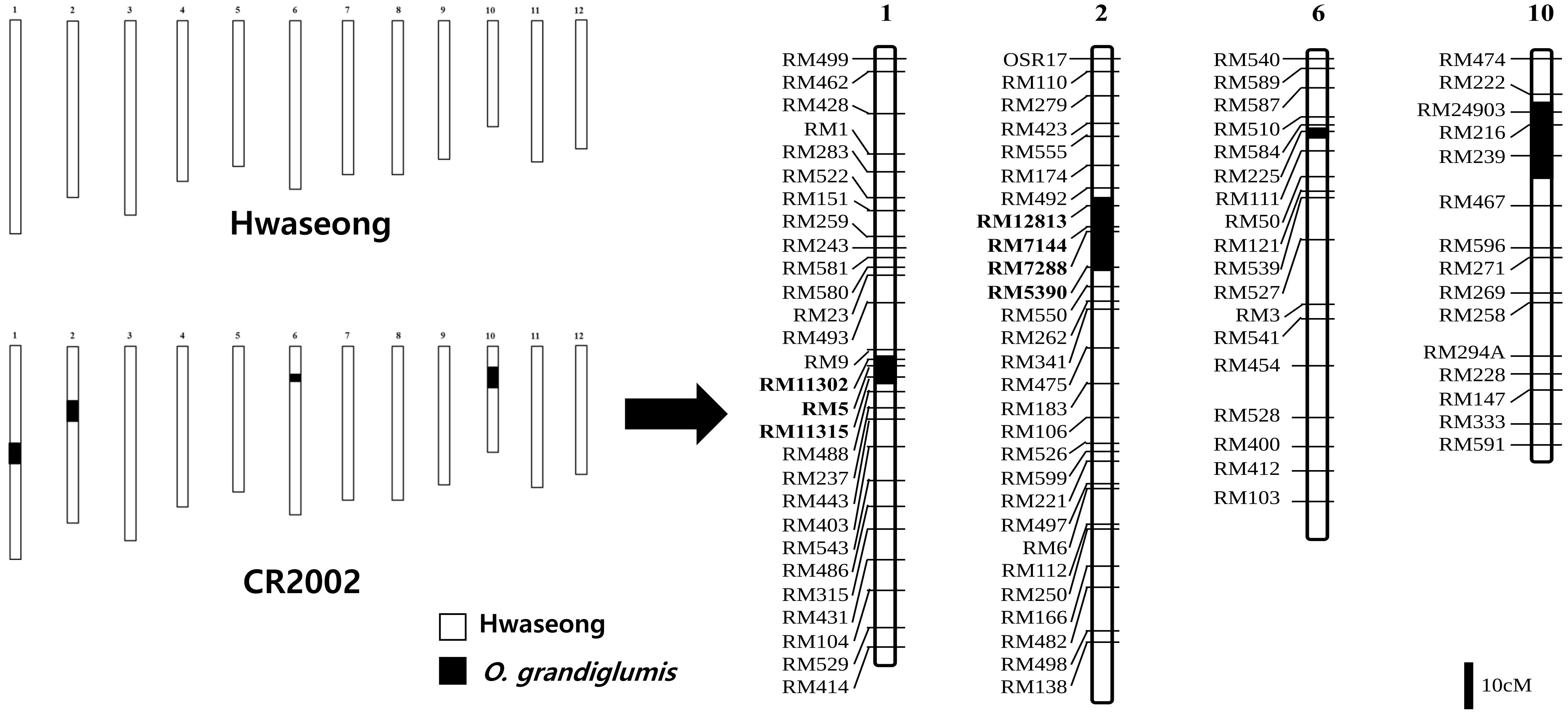

Supplementary figure 1. Graphical genotypes of plant materials. Black boxes with SSR markers mark the *O. grandiglumis* segments introgressed into *O. sativa cv.* Hwaseong. Chromosomes 3, 4, 5, 7, 8, 9, 11 and 12 have no introgression. To represent the detailed graphical genotype information of CR2002, the genetic map on the right side is used from Yoon et al. (2006) [41] with modifications.

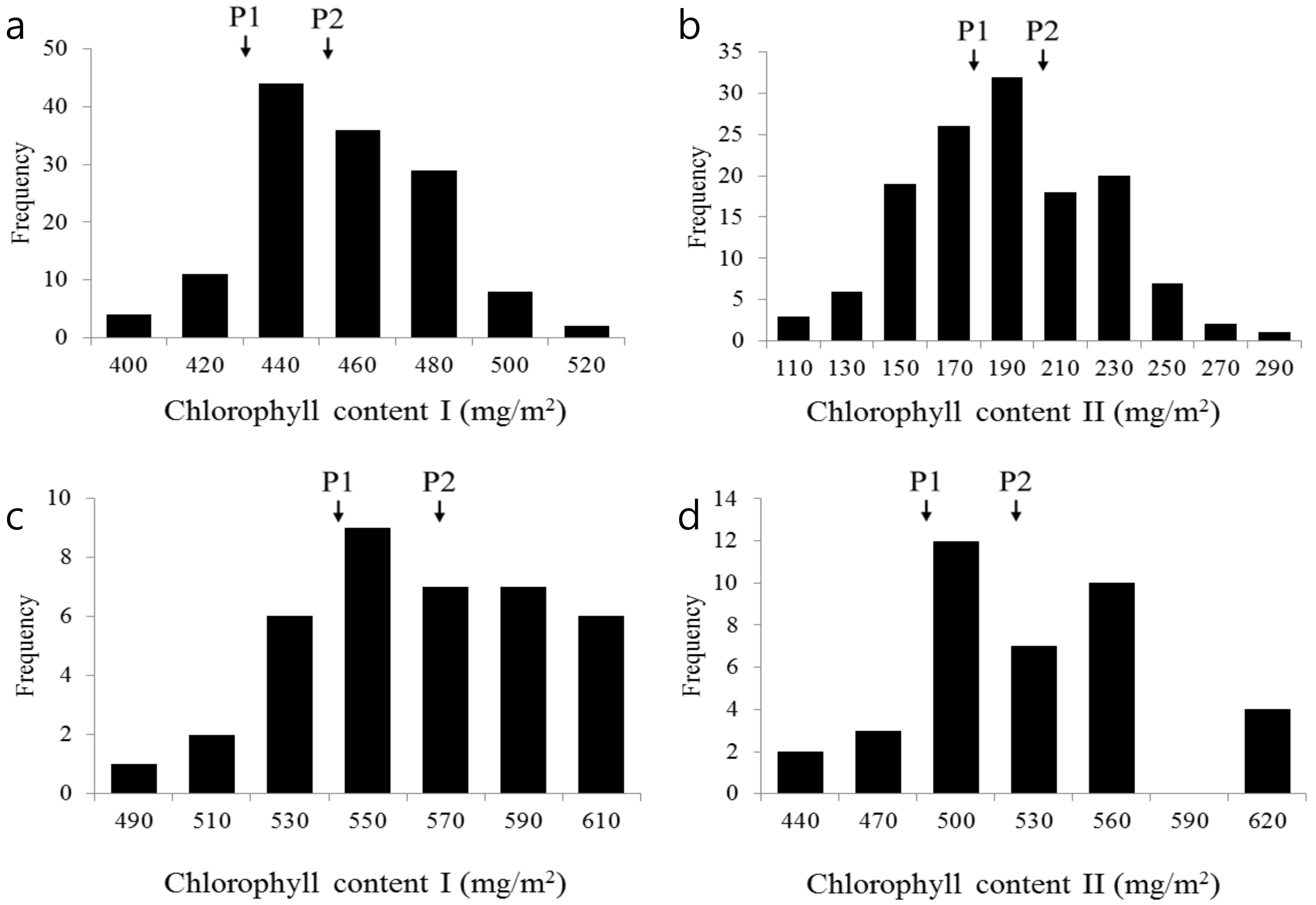

Supplementary figure 2. Frequency distribution of chlorophyll contents in the F_3 (a, b) and F_4 populations (c, d). Chlorophyll contents were evaluated using CCM-300 (OPTI-SCIENCE) at heading stage (chlorophyll content I) and one month after heading (chlorophyll content II). P1: Hwaseong, P2: CR2002

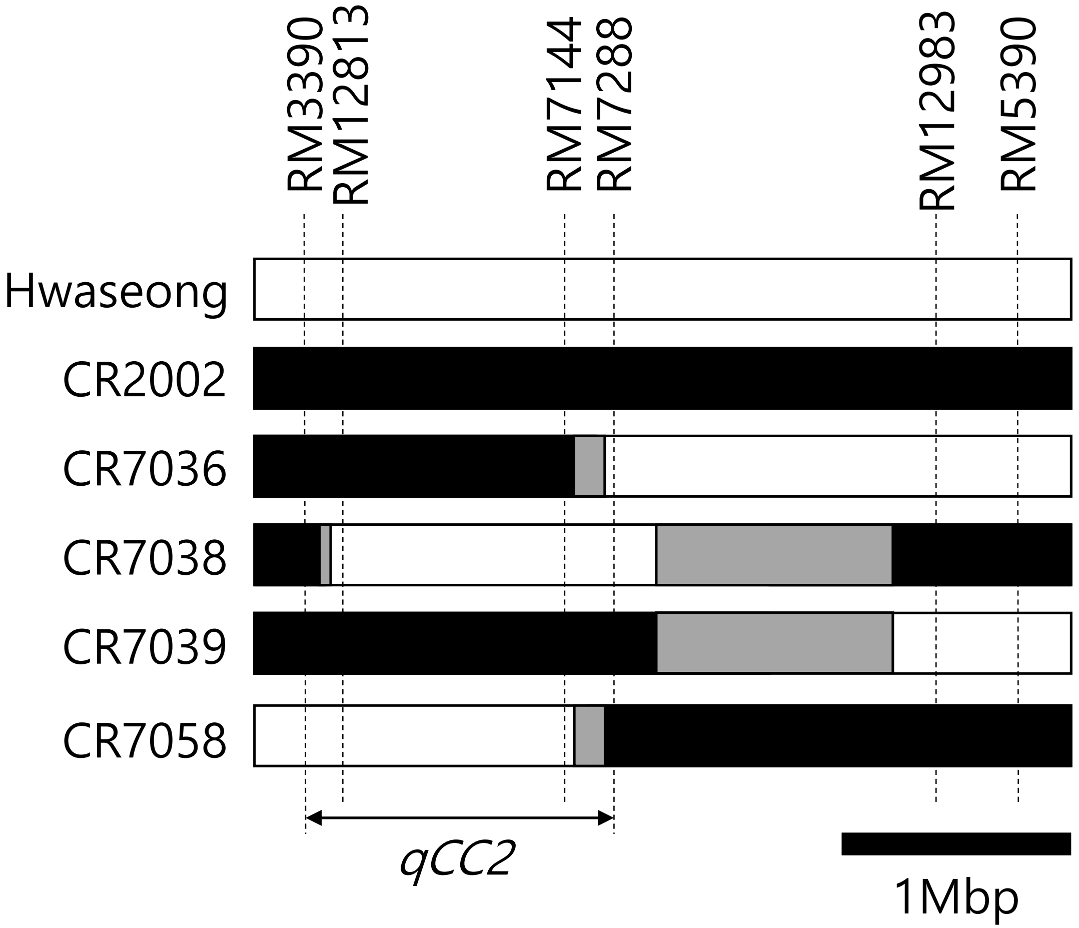

	SPAD			
	95 DAT[1)]	105 DAT	115 DAT	125 DAT
Hwaseong	36.9±2.5 b	32.5±2.6 b	26.1±3.6 cd	20.3±4.5 bc
CR2002	40.7±1.9 a	37.9±3.2 a	32.3±3.5 ab	27.5±4.5 a
CR7036	41.1±0.8 a	36.9±1.9 a	29.1±2.5 bc	24.6±3.1 ab
CR7038	36.6±1.5 b	32.2±1.6 b	26.3±1.6 cd	19.1±3.1 bc
CR7039	41.9±0.8 a	39.0±2.0 a	33.9±2.6 a	27.0±3.5 a
CR7058	36.3±2.2 b	29.8±2.7 b	24±3.3 d	18.5±4.4 c

□ Hwaseong homozygous ■ *O. grandiglumis* homozygous ▨ Recombination break point

Supplementary figure 3. Substitution mapping of the *qCC2* region using five ILs with different recombination break point. Data are represented as mean ± standard deviation. Means followed by the same letter in each column are not significantly different among the groups at P = 0.05 based on Tukey's test. [1)]DAT: days after transplanting. Black, blank, and gray regions mark the *O. grandiglumis*-specific, the Hwaseong genomic regions, and recombination break point, respectively. Based on the genotypes of ILs, the *qCC2* was delimited between two SSR markers, RM3390 and RM7288.

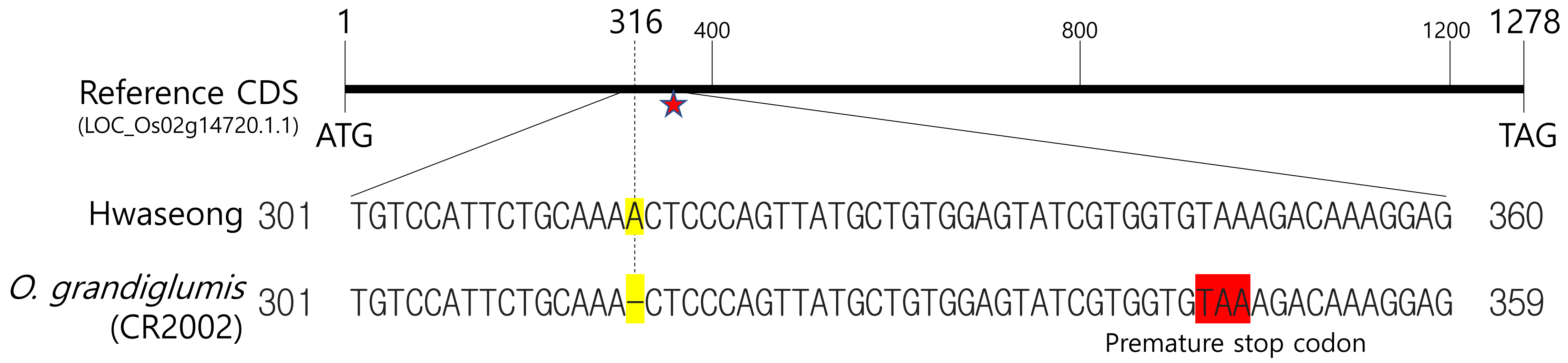

Supplementary figure 4. Comparison of *GW2* coding sequence (CDS) between Hwaseong and *O. grandiglumis* (CR2002). Red star indicates location of stop codon.

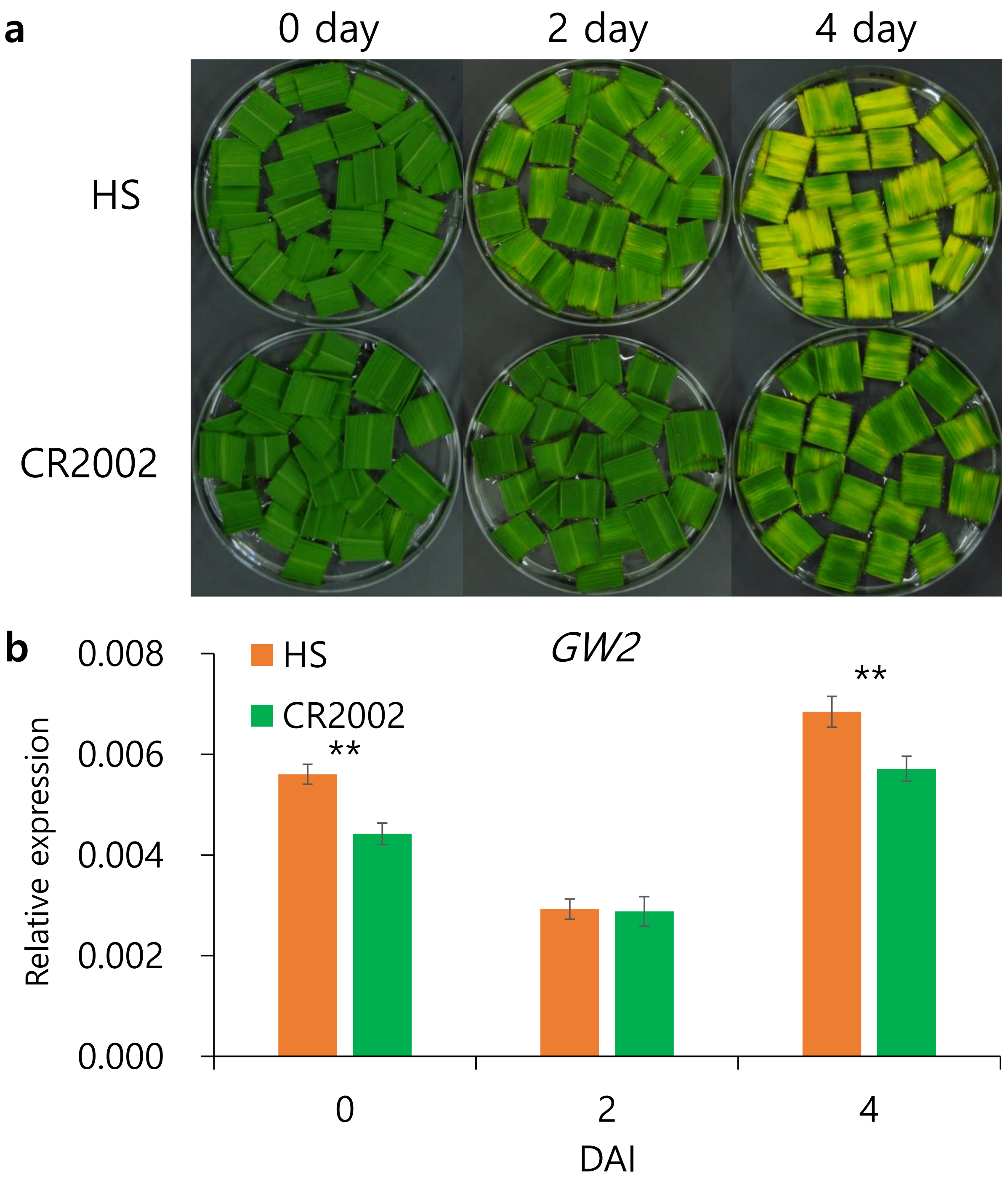

Supplementary figure 5. (**a**) Dark-induced senescence of Hwaseong and CR2002. Comparison of gene expression pattern of *OsGW2* during the dark-induced senescence. Transcript level of genes was determined by qRT-PCR with *OsUBQ5* used for normalization. ** indicates significant difference at P < 0.01 based on Student's *t*-test. DAI: days after incubation.

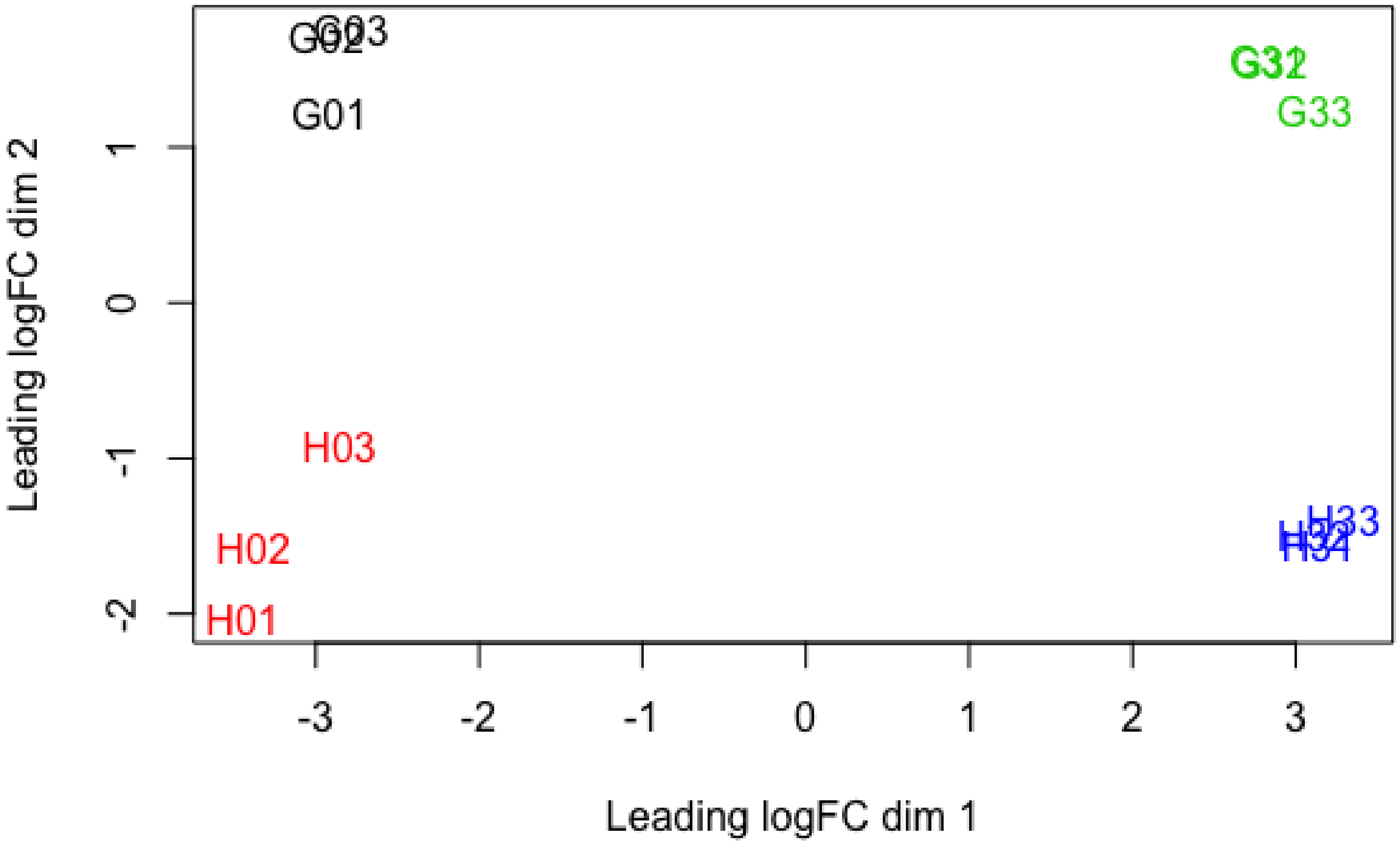

Supplementary figure 6. Multi-dimensional scaling of the RNA sequencing data. The samples (three biological replicates) of each treatment were projected in four principal components; duplicates are clustered together, indicating that the replicates were consistently generated.

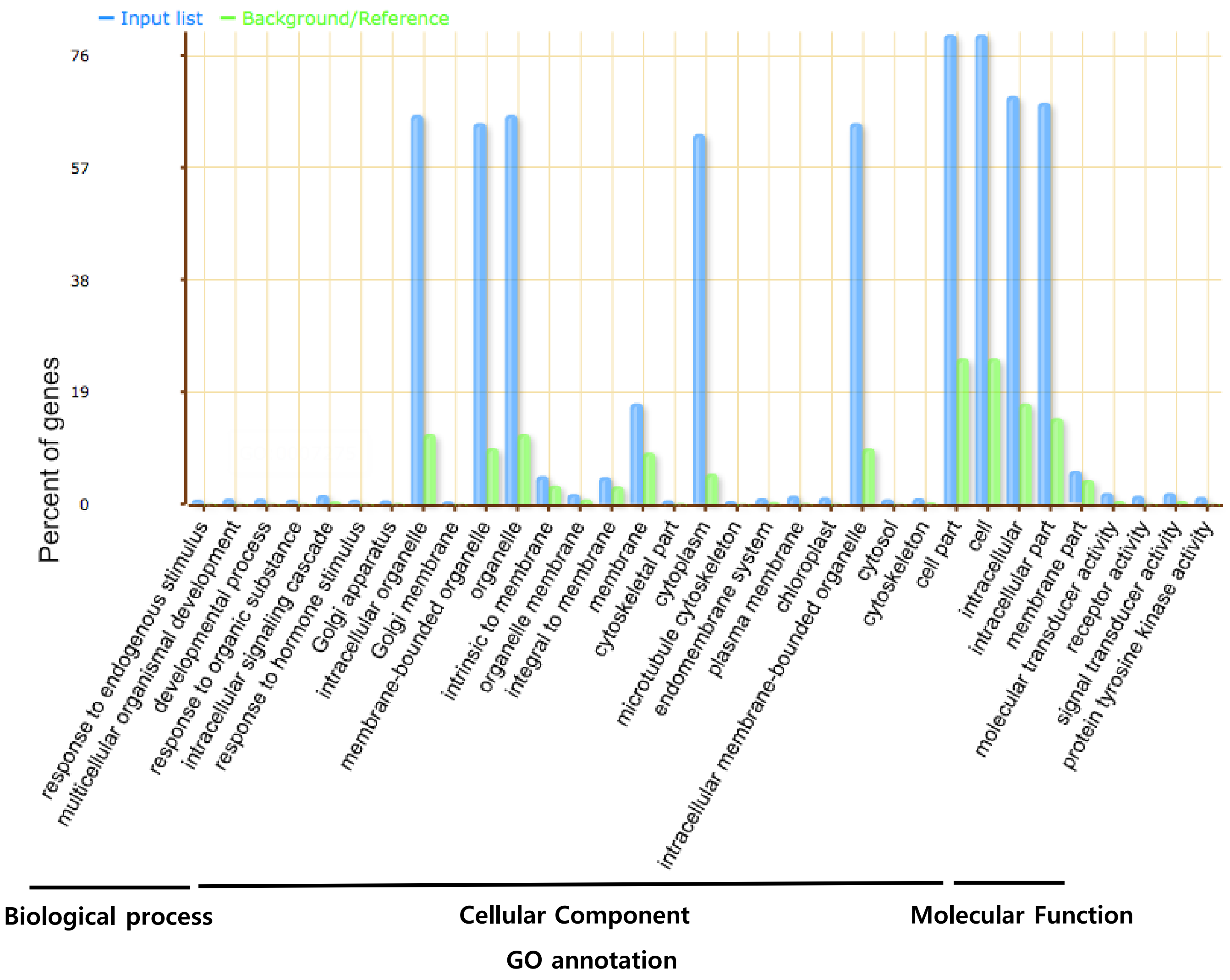

Supplementary figure 7. Significantly enriched GO terms for Hwaseong-specific DEGs. Blue and green bars indicate input list of Hwaseong and background/reference, respectively.

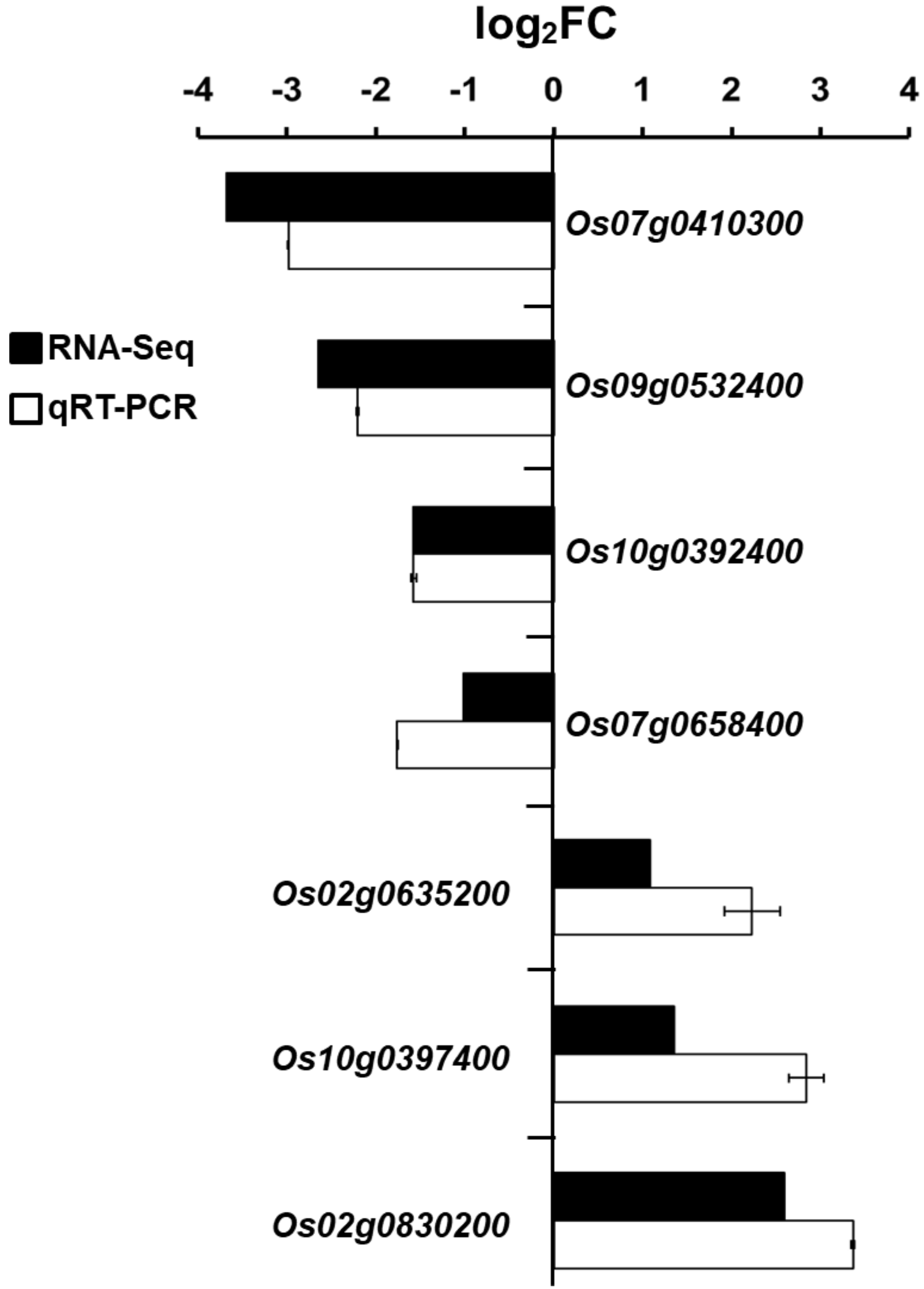

Supplementary figure 8. Validation of gene expression levels between RNA-seq and qRT-PCR. Change of gene expression levels of seven DEGs specifically identified in CR2002 was confirmed by qRT-PCR. *OsTCTP* was used for normalization. Error bars indicate the standard deviation of three replications.
